# Oral Manifestations: A Reliable Indicator for Undiagnosed Diabetes Mellitus Patients

**DOI:** 10.1055/s-0042-1755553

**Published:** 2022-10-11

**Authors:** Maliha Shahbaz, Farhat Kazmi, Hanna Abdul Majeed, Saadia Manzar, Faiza Awais Qureshi, Shahrayne Rashid

**Affiliations:** 1 Department of Oral Biology, Lahore Medical and Dental College, Lahore, Pakistan; 2 Department of Oral Pathology, Rashid Latif Dental College/Rashid Latif Medical Complex, Lahore, Pakistan; 3 Department of Operative Dentistry, Rashid Latif Dental College/Rashid Latif Medical Complex, Lahore, Pakistan; 4 Department of Oral & Maxillofacial Surgery, Rashid Latif Dental College/Rashid Latif Medical Complex, Lahore, Pakistan; 5 Department of Community Dentistry, Rashid Latif Dental College/Rashid Latif Medical Complex, Lahore, Pakistan

**Keywords:** diabetes, prediabetes, HbA1c, fissured tongue, tongue manifestations

## Abstract

**Objectives** This article identifies undiagnosed DM (UDM) cases in the Pakistani population by perceiving the signs and symptoms of DM and associating them with oral manifestations.

**Material and Methods** In this cross-sectional study, patients showing at least three or more classical or warning signs like polydipsia, polyuria, polyphagia, and general weakness were considered UDM cases. Detailed oral examination for gingivitis, periodontitis, halitosis, xerostomia, and tongue manifestations was done followed by the hemoglobin A1c (HbA1c) analysis.

**Results** Out of 5,878 patients, 214 UDM cases were identified, where 31.8% and 39.7% of the patients were diagnosed as prediabetics and diabetics, respectively, based on HbA1c analysis. Prevalence of gingivitis (97.6%), fissured tongue (91.8%), generalized periodontitis (85.9%), thick saliva (87.1%), xerostomia (84.7%), burning mouth syndrome (63.5%), yellow discoloration of tongue (57.6%), and ecchymosis/ulcers (43.5%) were more in diabetics as compared to prediabetic patients and normal population.

**Conclusion** The oral manifestations can be crucial for identifying UDM cases. Dentists can play a pivotal role by taking detailed history and thorough oral examination. If three or more symptoms as concluded above are present, an HbA1c analysis should be conducted to prevent preop and postop complications associated with DM.

## Introduction

Diabetes mellitus (DM) is a growing public health concern worldwide. 1 2 It can have profound long-term implications on the lives and well-being of individuals, families, and society. 1 It is a chronic metabolic disorder, and it is of two types: type 1 (also known as insulin-dependent) and type 2 (also known as noninsulin-dependent). 3 Type 2 DM represents approximately 95% of diabetic cases. 4

DM affects all age groups but is more common in adults. According to the International Diabetes Federation, globally, its prevalence has increased dramatically over the past few years, and it is expected to increase threefold in the next decade. 1 It is also estimated that 49.7% of people living with type 2 diabetes are undiagnosed. 5 In Pakistan, the prevalence of type 2 DM is approximately 16.98%, whereas the prevalence of prediabetes is 10.91%. 6 7 According to literature, around two-thirds of prediabetes tend to manifest DM in 3 years' time span. 8 9 A delay of 2 to 7 years has been estimated by studies between onset of diabetes myelitis and its definitive diagnosis. 10

The common risk factors for type 2 DM include family history of diabetes, age greater than 35 years, obesity, hypertension, sedentary lifestyle, and gestational diabetes. 4 However, people and doctors should be well aware of classical or warning symptoms like unexplained weight loss, polydipsia, polyuria, polyphagia, fatigue, irritability, dry mouth, and burning pain or numbness of feet. 4 DM ultimately leads to comorbid symptoms like microvascular (nephropathy, neuropathy, retinopathy) or macrovascular (cardiovascular and cerebrovascular) manifestations. 11 Several conditions that deter oral health have also been associated with DM, namely periodontal problems, xerostomia, halitosis, fungal infections, caries, neurosensory dysesthesia, and oral mucosal lesions (like angular cheilitis, stomatitis, geographic, and fissured tongue). 3 12 13 14

DM is directly or indirectly associated with cardiovascular and cerebrovascular or infectious diseases. It has a high mortality rate and is considered to be one of the leading causes of death worldwide. 15 It is estimated that one in two people with DM are unaware of their health status. 1 This is because often, diabetes has a latent, asymptomatic phase of subclinical stage that goes undiagnosed for several years. 15 This applies to Pakistan as well; a comprehensive overview of the Pakistani health care system suggests a high burden of undiagnosed DM (UDM) cases, approximately 1 in 8,487, which estimates to be 3 million. 1 16 If the current trend of UDM continues then, Pakistan is likely to achieve the highest prevalence of DM worldwide. 17 In many UDM patients, vascular complications are already present, and they are unaware of these complications. 5 All these factors can play an adverse role in planning their dental treatment as most dental practitioners depend only on the history given by the patients 16 18 because such patients during dental treatment are more prone to develop hypoglycemia and require comprehensive management of oro-dental infections. 19

Individuals with DM are more vulnerable to develop oral manifestations. 8 19 20 Thus, the present study was conducted to identify UDM cases in the Pakistani population by perceiving the signs and symptoms of DM and associating these symptoms with oral manifestations. Dentists can play an active role in identifying and reducing the associated morbidity and mortality with UDM by evaluating their patients' oral health so that such patients can be timely referred to physicians for definitive diagnosis.

## Material and Methods

The present cross-sectional study was conducted on the Pakistani population at tertiary care hospital setting, from October 1, 2020 to April 1, 2021. Ethical approval was obtained from the ethical review committee of hospital with IRB no: 2520. A total of 5,878 patients presented to the outpatient department, the study included both genders, with age ranging from 25 to 75 years. All those patients who gave a history of classical or warning signs like polydipsia, polyuria, polyphagia, 4 and general weakness were included in the study.

Apart from these classical signs, history was also taken about unexplained weight loss, tingling sensation, and numbness of hands and feet. Unintentional loss of more than 5% of normal body weight, or more than 10 lbs (4.5 kg) in 6 to 12 months or less was considered as unexplained weight loss. Whereas, presence of unusual prickling sensations in the lower limbs as sharp, stabbing, lancinating, or electric shock like, was considered as numbness and tingling in the extremities.

Risk factors such as obesity and family history of DM were also taken into consideration. Known DM patients, immunocompromised, and mentally or physically disabled patients were excluded from the study.

Informed consent was taken before history taking and oral examination. Patients with a history of three or more classical DM signs were assumed as UDM patients and were referred for clinical oral examination and hemoglobin A1c (HbA1c) analysis. Clinical examination was performed using sterile examination gloves, mouth mirror, periodontal probe, sterile gauze, and wooden tongue depressor under dental unit light.

**Fig. 1:**
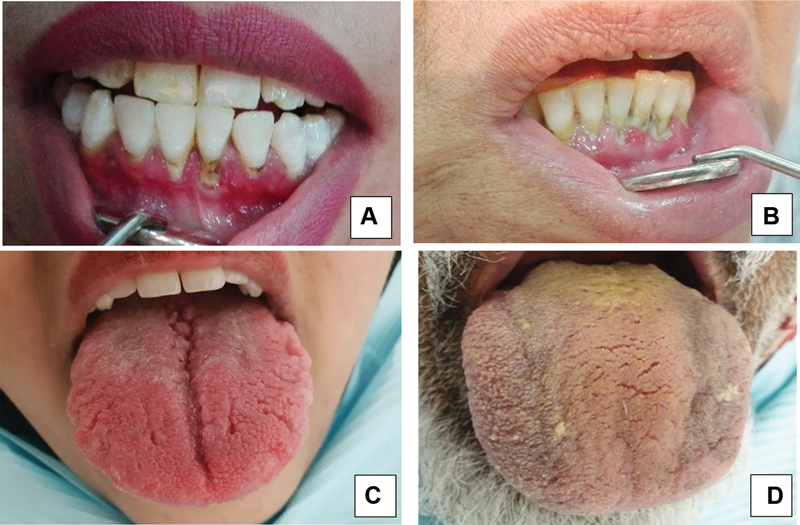
Clinical pictures of patients with ( **A** ) localized periodontitis, ( **B** ) generalized periodontitis, ( **C** ) fissured tongue, and ( **D** ) yellow tongue coating along with fissured tongue.

Parameters for evaluating oral manifestations were:

- Gingivitis – the presence of bleeding on probing.
- Periodontitis – clinical attachment loss:
  Stage I: 1–2 mm
  Stage II: 3–4 mm
  Stage III: ≤ 5 mm
  Stage IV: ≥ 5 mm
o Localized (less than 30% sites involving incisors and first molar only) ( Fig. 1A ).
o Generalized (more than 30% with three teeth other than incisors and first molars involved) ( Fig. 1B ). 21
- Halitosis – evaluated through the history of bad breath.
- Xerostomia – evaluated when dental mirror was sticking to the tongue or buccal mucosa (either no saliva pooling or thick ropy saliva) and burning mouth syndrome (BMS).
- Tongue manifestations assessed were fissured tongue, presence of ulcerations, and color of tongue coating (white, pale yellow, or yellow) ( Fig. 1C and D ).

### Assessing the Blood Glucose Levels

According to the American Diabetic Association (ADA), HbA1c is a reliable diagnostic test for diabetes and prediabetes. 22 23 HbA1c is a blood test that measures the average blood sugar (glucose) attached to hemoglobin over the past 3 months. 22 A high HbA1c level is indicative of diabetes. 22 Thus, interpretation of HbA1c levels was made according to the ADA guidelines; subjects with HbA1c levels within the range of 4.0 to 5.6% were nondiabetic patients (regular patients), those between 5.7 and 6.4% were prediabetic. In contrast, those with HbA1c levels greater than 6.5% were diagnosed as diabetic patients. 23 24

### Data Analysis

The data was entered and analyzed using the Statistical Package of Social Sciences (SPSS) version 24.0. Mean and standard were calculated for quantitative variables like age and HbA1c. Chi-square test was used to explore the significant association between diabetic status and classical signs, including oral manifestations. A *p* -value of ≤ 0.05 was considered significant.

## Results

**Fig. 2:**
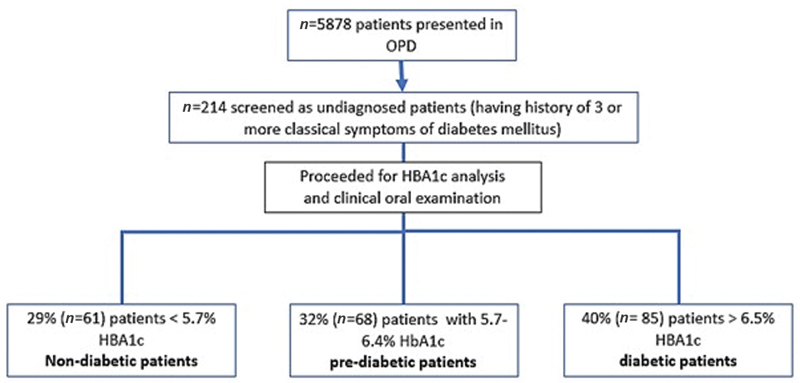
Flowchart of the study protocol.

Out of 5,878 patients, 214 (3.6%) patients were identified as UDM who gave history of three or more classical signs of DM ( Fig. 2 ). These patients were proceeded for oral examination and HbA1c analysis. According to the ADA criteria, out of these 214 UDM patients, 28.5% ( *n* = 61) were nondiabetic, 31.8% ( *n* = 68) patients were found to be prediabetic, and 39.7% ( *n* = 85) were identified as diabetic patients ( Fig. 2 ).

The mean age of participants was almost 50 ± 8 years, and the mean value of HbA1c was 6.6 with a standard deviation of 1.45. Table 1 shows that the mean age of nondiabetic and prediabetic participants was similar (47 ± 5 years), whereas the mean age of diabetic patients was 54 ± 9 years. The mean value of HbA1c among nondiabetic, prediabetic, and diabetic participants was 5.23 ± 0.20, 6.00 ± 0.21, and 8.01 ± 1.25, respectively ( Table 1 ).

**Table 1:** Description of age and HbA1c by classification of diabetic status

Diabetic status	Variable name	*N*	Minimum	Maximum	Mean	Standard deviation
Nondiabetic	Age	61	39	61	47.07	5.205
Prediabetic		68	37	61	47.12	5.307
Diabetic		85	38	88	54.29	9.396
Nondiabetic	HbA1c	61	4.80	5.60	5.2344	.20321
Prediabetic		68	5.70	6.40	6.0015	.21682
Diabetic		85	6.50	11.00	8.0153	1.25247

Classical diabetic signs were slightly more prevalent in female participants; however, the results revealed insignificant *p* -value for both genders, showing that they had the same chances to develop DM ( Table 2 ).

**Table 2:** Association of classical signs of diabetes mellitus and diabetic status

Variable name	Category name	Frequency (%)	Diabetic status			*p* -Value
			Nondiabetic	Prediabetic	Diabetic	
Gender	Male	*N* (%)	30 (49.2)	30 (44.1)	41 (48.8)	0.803
	Female	*N* (%)	31 (50.8)	38 (55.9)	43 (51.2)	
Polydipsia	No	*N* (%)	0	0	0	0.000
	Yes	*N* (%)	61 (100)	68 (100)	85 (100)	
Polyurea	No	*N* (%)	0	0	0	0.000
	Yes	*N* (%)	61 (100)	68 (100)	85 (100)	
Polyphagia	No	*N* (%)	49 (80.3)	0	0	0.000
	Yes	*N* (%)	12 (19.7)	68 (100)	85 (100)	
Numbness	No	*N* (%)	61 (100)	66 (97.1)	0	0.000
	Yes	*N* (%)	0	2 (2.9)	85 (100)	
Unexplained weight loss	No	*N* (%)	31 (50.8)	7 (10.3)	0	0.000
	Yes	*N* (%)	30 (49.2)	61 (89.7)	85 (100)	

All 214 patients gave a history of polydipsia and polyuria; however, polyphagia was predominant among prediabetic and diabetic patients ( Table 2 ). Most diabetic patients suffered from numbness and tingling in their extremities, compared with prediabetic and nondiabetic patients ( Table 2 ). A significant number of prediabetic and diabetic participants had a history of more than 10 lbs (4.5 kg) of unexplained weight loss in 6 to 12 months compared with nondiabetic patients ( Table 2 ).

The percentage of oral manifestations recorded in UDM patients is depicted in Table 3 , where generalized periodontitis, xerostomia, and BMS, with yellow discoloration of tongue were dominant in diabetics compared with normal and prediabetic patients.

**Table 3:** Association of oral manifestations of diabetes mellitus and diabetic status

Variable name	Category name	Frequency (%)	Diabetic status			*p* -Value
			Nondiabetic	Prediabetic	Diabetic	
Halitosis	No	*N* (%)	53 (86.9)	8 (11.8)	2 (2.4)	0.000
	Yes	*N* (%)	8 (13.1)	60 (88.2)	83 (97.6)	
Gingivitis	No	*N* (%)	40 (65.5)	8 (11.8)	2 (2.4)	0.000
	Yes	*N* (%)	21 (34.4)	60 (88.2)	83 (97.6)	
Localized periodontitis	No	*N* (%)	53 (86.9)	13 (19.1)	74 (87.1)	0.000
	Yes	*N* (%)	8 (13.1)	55 (80.9)	11 (12.9)	
Generalized periodontitis	No	*N* (%)	56 (91.8)	56 (82.4)	12 (14.1)	0.000
	Yes	*N* (%)	5 (8.2)	12 (17.6)	73 (85.9)	
H/O dry mouth	No	*N* (%)	61 (100)	57 (83.8)	13 (15.3)	0.000
	Yes	*N* (%)	0	11 (16.2)	72 (84.7)	
Burning mouth syndrome	No	*N* (%)	61 (100)	61 (89.7)	31 (36.5)	0.000
	Yes	*N* (%)	0	7 (10.3)	54 (63.5)	
Tongue color	White	*N* (%)	58 (95.1)	52 (76.5)	11 (12.9)	0.000
	Pale Yellow	*N* (%)	3 (4.9)	12 (17.6)	25 (29.4)	
	Yellow	*N* (%)	0	4 (5.9)	49 (57.6)	
Tongue fissure	No	*N* (%)	61 (100)	63 (92.6)	7 (8.2)	0.000
	Yes	*N* (%)	0	5 (7.4)	78 (91.8)	
Ecchymosis/Ulcers	No	*N* (%)	61 (100)	62 (91.2)	48 (56.5)	0.000
	Yes	*N* (%)	0	6 (8.8)	37 (43.5)	
Consistency of saliva	Thick ropy	*N* (%)	3 (4.9)	12 (17.6)	74 (87.1)	0.000
	Thin	*N* (%)	58 (95.1)	56 (82.4)	11 (12.9)	

Other tongue manifestations like fissured tongue and ecchymosis/ulcers with thick saliva (stringy or rope-like) were observed more in diabetic patients than prediabetic and nondiabetic participants ( Table 3 ).

## Discussion

The prevalence of type 2 diabetes and prediabetes has been increasing rapidly in developing countries, particularly in Pakistan. 1 7 25 Comprehensive strategies regarding awareness of warning signs, timely diagnosis, and treatment planning of DM are lacking. 6 7 17

Type 2 DM which accounts for 85 to 95% of all diabetes, has a latent, asymptomatic period of subclinical stages which often remain undiagnosed for several years. 1 During this period of undiagnosed disease, risk factors for diabetic micro- and macrovascular complications are markedly elevated in the background of diabetic disease progression. Thus, in many Asian patients, warning signs and vascular complications are already present at the time of diagnosis of diabetes. 4 Accordingly, it was observed in our study, that among the 214 UDM cases, 71.5% participants were prediabetic and diabetic. The prevalence of UDM was almost the same in both genders; however, previous demographic data shows that old-aged females are most likely to be affected by DM. 7 13 24

According to literature, patients with DM are more prone to develop oral manifestations, 8 19 20 and are more susceptible to develop periodontal diseases. 26 Consequently, in the current study, majority of the patients with higher HbA1c levels showed generalized periodontitis. These findings are in accordance with earlier studies 27 28 showing that people with type 2 DM are three times more likely to develop periodontal diseases than people without DM. 27 28 This is due to the fact that status of periodontal disease in uncontrolled diabetic patients is influenced by glycemic control, increased production of advanced glycated end-products, and poor glycemic control cause oxidative stress to the gingiva, which in turn lead to periodontal disease. 29 30

The risk of halitosis (bad breath) increases with elevated levels of HbA1c 31 In the present study, around 88% of prediabetic and 97% of diabetic patients presented with halitosis which is in accordance with studies done by Choi and Al-Zahrani et al showing a strong association between halitosis and elevated levels of HbA1c. 31 32

Xerostomia is regarded as one of the most prevalent signs of DM. 13 In the present study, xerostomia was the fifth most common oral manifestation (after gingivitis, periodontitis, halitosis, and fissure tongue), conforming with literature. 2 3 13 20 The presence of thick ropy saliva is also a common feature with higher HbA1c levels; in our study, 87% of diabetic people presented with thick ropy saliva, which is in line with work done by Al-Maskari et al and Chávez et al. 3 33 Xerostomia causes dryness which disturbs the delicate lining of oral mucosa leading to BMS. 3 34 Our study data showed that around 63% of the participants had BMS, which corresponds with Gurvits and Tan. 35 Prevalence of BMS was lesser in diabetic and prediabetic patients probably because it has multifactorial etiologies other than poor glycemic control, such as chronic stress disorder, dietary and prosthesis allergies, angiopathy, candidiasis, and regional neuropathy. 34 36

Oral cavity mirrors the general health status of a person and supports the relationship between oral health and DM, hence examination of the tongue plays a vital role in the identification and prognosis of DM. 20 In the present study, the tongue manifestations taken into consideration were fissured tongue, ulceration, and color of tongue coating. Fissures develop on the dorsal surface of the tongue due to inadequate glycemic control, immunological changes, alteration in microcirculation, and salivary flow. 34 37 Manifestations like fissure tongue were prevalent in patients with higher HbA1c levels, accounting to 91% of diabetic patients. The findings of our study are consistent with the results of the study done on the Japanese population by Hsu et al. 20

Furthermore, in the current study, there was a greater prevalence of both yellow (57.6%) and pale yellow (29.4%) tongue coatings in patients having high levels of HbA1c. Similar findings regarding tongue coatings are observed by Tomooka et al in prediabetic and diabetic Japanese patients. 20 38 However, the prevalence of ulceration on the tongue was relatively lower (around 44%) in diabetic patients, which is in accordance with previous studies. 20 39

## Conclusion

Our study identified all UDM patients, on the basis of history and oral examination, further confirmed by HbA1c, and were categorized as prediabetics and diabetics based on HbA1c levels. Overall, these diabetic patients had poor glycemic control since they were unaware of their medical condition, and were neither taking hypoglycemics nor were on dietary restrictions. The results of the current study reveal that fissure tongue, halitosis, generalized periodontitis, tongue coatings, thick ropy saliva, and xerostomia were more prevalent in UDM due to poor glycemic control as compared with nondiabetics. These oral signs and symptoms can be crucial for identifying UDM cases. Dentists can play an essential role by performing thorough oral examination. If three or more symptoms as concluded above are present, an HbA1c test should be conducted. Moreover, extra precautions should be considered, especially during invasive procedures. They should strictly adhere to aseptic techniques, minimize iatrogenic tissue injury, and access the need for prophylactic antibiotics to avoid postoperative infections.

## Limitations

Sample size was limited, large-scale studies should carried out. Further, such studies should be conducted on different populations to validate the findings.

## Future Recommendations

Practice of conducting HbA1c tests should be implemented in all patients identified with three or more established oral manifestations. All private and government dental institutes throughout the country should focus on diabetic awareness programs, which will help save millions from morbidity and mortality secondary to DM.

## References

[1] IDF Diabetes Atlas Committee Saeedi P Petersohn I Salpea P Global and regional diabetes prevalence estimates for 2019 and projections for 2030 and 2045: results from the International Diabetes Federation Diabetes Atlas, 9th edition Diabetes Res Clin Pract 2019 157 107843 31518657 10.1016/j.diabres.2019.107843

[2] Ali Hassan S Pratyusha F Diabetes and oral diseases- a review IP J Nutr Metab Heal Sci. 2020 3 01 6 9

[3] Al-Maskari A Y Al-Maskari M Y Al-Sudairy S Oral manifestations and complications of diabetes mellitus: a review Sultan Qaboos Univ Med J 2011 11 02 179 186 21969888 PMC3121021

[4] Ramachandran A Know the signs and symptoms of diabetes Indian J Med Res 2014 140 05 579 581 25579136 PMC4311308

[5] Akhtar S Nasir J A Abbas T Sarwar A Diabetes in Pakistan: a systematic review and meta-analysis Pak J Med Sci 2019 35 04 1173 1178 31372163 10.12669/pjms.35.4.194 PMC6659044

[6] Ijaz M Ali I Hussain A Diabetes mellitus in Pakistan: the past, present, and future Int J Diabetes Dev Ctries 2020 40 01 153 154

[7] Aamir A H Ul-Haq Z Mahar S A Diabetes Prevalence Survey of Pakistan (DPS-PAK): prevalence of type 2 diabetes mellitus and prediabetes using HbA1c: a population-based survey from Pakistan BMJ Open 2019 9 02 e025300 10.1136/bmjopen-2018-025300 PMC6398762 30796126

[8] Genco R J Borgnakke W S Diabetes as a potential risk for periodontitis: association studies Periodontol 2000 2020 83 01 40 45 32385881 10.1111/prd.12270

[9] Centers for Disease Control and Prevention . “National Diabetes Statistics Report, 2020.” 2020.https://www.cdc.gov/diabetes/pdfs/data/statistics/national-diabetes-statistics-report.pdf. Accessed February 3, 2022

[10] Heji E S Bukhari A A Bahammam M A Homeida L A Aboalshamat K T Aldahlawi S A Periodontal Disease as a predictor of undiagnosed diabetes or prediabetes in dental patients Eur J Dent 2021 15 02 216 221 33285572 10.1055/s-0040-1719208 PMC8184281

[11] Stumvoll M Goldstein B J van Haeften T W Type 2 diabetes: principles of pathogenesis and therapy Lancet 2005 365(9467):1333 1346 15823385 10.1016/S0140-6736(05)61032-X

[12] Indurkar M S Maurya A S Indurkar S Oral manifestations of diabetes Clin Diabetes 2016 34 01 54 57 26807010 10.2337/diaclin.34.1.54 PMC4714722

[13] Moosa Y Shahzad M Shaikh A A Matloob S A Khalid M. Influence of diabetes mellitus on oral health Pak Oral Dent J 2018 38 01 67 70

[14] Grigoriadis A Koutounidou S Räisänen I Arsenakis M Sakellari D Interaction between TCF7L2 rs7903146 genotype, HbA1c levels, and the periodontal status of dental patients Eur J Dent 2021 15 03 495 501 34041727 10.1055/s-0041-1725578 PMC8382464

[15] Lin X Xu Y Pan X Global, regional, and national burden and trend of diabetes in 195 countries and territories: an analysis from 1990 to 2025 Sci Rep 2020 10 01 14790 32901098 10.1038/s41598-020-71908-9 PMC7478957

[16] Mehmood K Junaid N Prevalence of undiagnosed type 2 diabetes mellitus in Pakistan: results of screen-diabetes disease registry J Pak Med Assoc 2018 68 08 1171 1178 30108381

[17] Hussain A Ali I Diabetes mellitus in Pakistan: a major public health concern Arch Pharm Pract (Mumbai) 2016 7 01 30 33

[18] Gazal G Management of an emergency tooth extraction in diabetic patients on the dental chair Saudi Dent J 2020 32 01 1 6 31920272 10.1016/j.sdentj.2019.07.004 PMC6950840

[19] Miller A Ouanounou A Diagnosis, management, and dental considerations for the diabetic patient J Can Dent Assoc 2020 86 k8 32543368

[20] Hsu P C Wu H K Huang Y C The tongue features associated with type 2 diabetes mellitus Medicine (Baltimore) 2019 98 19 e15567 31083226 10.1097/MD.0000000000015567 PMC6531228

[21] Tonetti M S Greenwell H Kornman K S Staging and grading of periodontitis: framework and proposal of a new classification and case definition J Periodontol 2018 89 01 S159 S172 29926952 10.1002/JPER.18-0006

[22] Sherwani S I Khan H A Ekhzaimy A Masood A Sakharkar M K Significance of HbA1c test in diagnosis and prognosis of diabetic patients Biomark Insights 2016 11 95 104 27398023 10.4137/BMI.S38440 PMC4933534

[23] American Diabetes Association Glycemic targets: standards of medical care in diabetes—2018. In: Diabetes Care. Vol. 41 American Diabetes Association 2018:S55–S64 10.2337/dc18-S006 29222377

[24] Gonzalez A Deng Y Lane A N Impact of mismatches in HbA _1c_ vs glucose values on the diagnostic classification of diabetes and prediabetes Diabet Med 2020 37 04 689 696 31721287 10.1111/dme.14181

[25] Meo S A Zia I Bukhari I A Arain S A Type 2 diabetes mellitus in Pakistan: current prevalence and future forecast J Pak Med Assoc 2016 66 12 1637 1642 27924966

[26] Kunsongkeit P Okuma N Rassameemasmaung S Chaivanit P Effect of vitamin C as an adjunct in nonsurgical periodontal therapy in uncontrolled type 2 diabetes mellitus patients Eur J Dent 2019 13 03 444 449 31280483 10.1055/s-0039-1693207 PMC6890498

[27] Vernillo A T Dental considerations for the treatment of patients with diabetes mellitus J Am Dent Assoc 2003 134(Spec No):24S 33S 18196670 10.14219/jada.archive.2003.0366

[28] Seethalakshmi C Reddy R C Asifa N Prabhu S Correlation of salivary pH, incidence of dental caries and periodontal status in diabetes mellitus patients: a cross-sectional study J Clin Diagn Res 2016 10 03 ZC12 ZC14 10.7860/JCDR/2016/16310.7351 PMC4843377 27134992

[29] Berniyanti T Wening G RS Palupi R Setyowati D Putri C R Low levels of tumor necrosis factor-? will prevent periodontitis exacerbation in type 2 diabetes mellitus Eur J Dent 2022 16 02 443 448 35016229 10.1055/s-0041-1739442 PMC9339937

[30] Vincent R R Appukuttan D Victor D J Balasundaram A Oxidative stress in chronic periodontitis patients with type II diabetes mellitus Eur J Dent 2018 12 02 225 231 29988233 10.4103/ejd.ejd_244_17 PMC6004793

[31] Choi J-S Association between self-assessed gingival bleeding and halitosis, and glycated hemoglobin levels in patients with diabetes J Korean Soc Dent Hyg 2020 20 01 19 27

[32] Al-Zahrani M S Zawawi K H Austah O N Al-Ghamdi H S Self reported halitosis in relation to glycated hemoglobin level in diabetic patients Open Dent J 2011 5 01 154 157 21966334 10.2174/1874210601105010154 PMC3178913

[33] Chávez E M Borrell L N Taylor G W Ship J A A longitudinal analysis of salivary flow in control subjects and older adults with type 2 diabetes Oral Surg Oral Med Oral Pathol Oral Radiol Endod 2001 91 02 166 173 11174593 10.1067/moe.2001.112054

[34] Cicmil S Mladenović I Krunić J Ivanović D Stojanović N Oral alterations in diabetes mellitus Balk J Dent Med 2018 22 01 7 14

[35] Gurvits G E Tan A Burning mouth syndrome World J Gastroenterol 2013 19 05 665 672 23429751 10.3748/wjg.v19.i5.665 PMC3574592

[36] Maltsman-Tseikhin A Moricca P Niv D Burning mouth syndrome: will better understanding yield better management? Pain Pract 2007 7 02 151 162 17559486 10.1111/j.1533-2500.2007.00124.x

[37] Hamrah MH, Baghalian A, Ghadimi S, et al. The prevalence and correlates of fissured tongue among outpatients in a regional area of Afghanistan: a cross-sectional study Clin Cosmet Investig Dent 2021 13 68 70 10.2147/CCIDE.S323428 PMC8325757 34345186

[38] Tomooka K Saito I Furukawa S Yellow tongue coating is associated with diabetes mellitus among Japanese non-smoking men and women: the Toon Health Study J Epidemiol 2018 28 06 287 291 29311441 10.2188/jea.JE20160169 PMC5976872

[39] El Toum S Cassia A Bouchi N Kassab I Prevalence and distribution of oral mucosal lesions by sex and age categories: a retrospective study of patients attending Lebanese School of Dentistry Int J Dent 2018 2018 4.030134E6 10.1155/2018/4030134 PMC5985080 29887889

